# The Effect of ZnO on the Failure of PET by Environmental Stress Cracking

**DOI:** 10.3390/ma13122844

**Published:** 2020-06-25

**Authors:** Ana Beatriz de Sousa Barros, Rômulo de Freitas Farias, Danilo Diniz Siqueira, Carlos Bruno Barreto Luna, Edcleide Maria Araújo, Marcelo Silveira Rabello, Renate Maria Ramos Wellen

**Affiliations:** 1 Department of Materials Engineering, Federal University of Paraíba, Cidade Universitária, João Pessoa 58051-900, PB, Brazil; bsbarros01@gmail.com; 2 Academic Unit of Materials Engineering, Federal University of Campina Grande, Av. Aprígio Veloso, 882 - Bodocongó, Campina Grande 58429-900, Paraíba, Brazil; romuloffarias@yahoo.com.br (R.d.F.F.); danilodinizsiqueira@gmail.com (D.D.S.); brunobarretodemaufcg@hotmail.com (C.B.B.L.); edcleide.araujo@ufcg.edu.br (E.M.A.); marcelo.rabello@ufcg.edu.br (M.S.R.)

**Keywords:** PET, PET/ZnO, NaOH, stress cracking, thermal and tensile properties

## Abstract

The aim of the present work is to evaluate the effect of NaOH solution as a stress cracking agent on the thermal and tensile properties of PET and PET/ZnO composites. The solutions were applied during tensile testing and the effects were monitored by differential scanning calorimetry (DSC), thermogravimetric analysis (TGA) and testing the actual mechanical properties. The rate of crystallization was increased when the samples were exposed to NaOH, as observed by both cold and melt crystallization; this is possibly due to the reduction in molar mass of the PET molecules caused by NaOH. During melting, the DSC peaks became more complex, which is probably due to the distinct macromolecular mass, as well as crystallites with different sizes and levels of perfection. According to TGA analyses, no drastic changes were observed on the thermal stability of PET due to the action of NaOH. The tensile properties were shown to decrease drastically upon exposure to NaOH, which is the main symptom of stress cracking, leading to increased fragility, as also observed in the scanning electron microscopy (SEM) images. The presence of ZnO improved PET crystallization and provided some protection against the harmful effects of NaOH.

## 1. Introduction

Poly (ethylene terephthalate) (PET) is a relatively low cost thermoplastic with excellent physical and mechanical properties, good processability and reasonable thermal stability, and ideal characteristics for packaging food products such as soft drinks, water and juices. PET is also widely used for durable articles in the electronics and automobile industries [1,2,3]. Despite these good properties, unexpected service failures in PET-based products have been reported [4], which, besides being a problem that plagues polymer engineers, have hindered the expansion of applications in areas considered critical, like aerospace components. When the causes of component failure have been identified, they were often related to isolated factors, such as residual stress, chemical attack, or thermal degradation. Among these causes, Environmental Stress Cracking (ESC) appears among the most frequent and is, therefore, highly important [5].

ESC is generally used to describe the product cracking phenomenon due to contact with aggressive fluids. This is not yet fully understood, but it happens when there is the simultaneous action of a chemical agent (liquid or vapor) and mechanical stress (external or internal), leading to cracking. It has been one of the most common causes of premature failure in plastic products, accounting for approximately 25% of reported cases [4,6]. Both amorphous and crystalline polymers show vulnerability to ESC, but the amorphous ones show greater susceptibility, possibly due to the larger free volume which facilitates chemical agent diffusion among the intermolecular regions [7]. Many ESC failures involve contact with fluids such as paints, adhesives, cleaning agents, aerosols, lubricants, and even food such as butter and ice cream [8].

In its various applications, PET may be subject to the simultaneous action of aggressive fluids and mechanical stresses and, hence, ESC investigation is fundamentally important. Studies on the stress cracking of PET were done with several types of fluids, including isopropanol, methanol, ethanol [9,10], and aqueous solutions of NaOH [8,9,10,11,12]. In the latter case, a reduction in molar mass was also observed during the experiments [11], and the action was faster in the amorphous regions [13]. Additionally, molar mass measurements performed on PET/clay composites showed that the clay accelerated the chemical attack on the PET matrix with higher concentrations of NaOH, i.e., 1M and 3M, but reduced the effect of mechanical stress on degradation. 

The properties of polymers can also be changed when inorganic fillers are added, which has been the focus of a number of publications, due to the diversified properties achieved in the final product. Among these additives, nanofillers may improve thermal stability, flame retardancy, the mechanical properties, vapor permeability, chemical resistance, and antimicrobial action [11]. As an optimized example, nanostructured zinc oxide (ZnO) has been used and good properties were obtained, such as low dielectric constants, high light transmittance, and antimicrobial activity [7,12,14]. No work has yet been done on the stress cracking behavior of PET/ZnO composites [15].

Based on the aforementioned observations, the present work investigates the ESC phenomenon in PET and PET/ZnO composites in contact with NaOH solutions in order to better understand their tensile and thermal behavior.

## 2. Experimental

### 2.1. Materials

Poly (ethylene terephthalate) CLEARTUF MAX, with an intrinsic viscosity of 0.84 dL/g, was supplied by M&G Chemicals as pellets. The active fluid was sodium hydroxide (NaOH), purchased from Labsynth Produtos para Laboratórios LTDA. The nanofiller was zinc oxide (ZnO), purchased from Acros Organics with purity 99.5% and a specific area of 28 m^2^/g. Raw materials were used as received without any further purification.

### 2.2. Compounding and Extrusion

**Figure 1:**
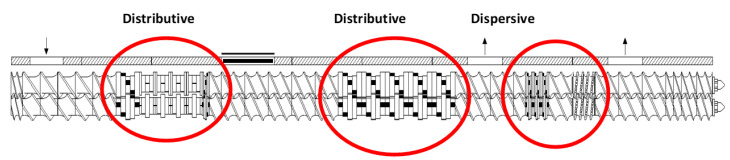
Extruder screw configured profile with distributive and dispersive mixing elements.

PET and ZnO were dried at 120 °C for 24 h prior to the mixing procedure. Firstly, a PET/ZnO masterbatch was prepared at a 7:1 ration using a Haake torque rheometer, operating with roller rotors at 260 °C, 60 rpm, for 10 min. The output was milled, and then compounded with PET in a Coperion Werner-Pfleiderer (Stuttgart-Feuerbach, Germany) model ZSK (D = 18 mm and L/D = 40) modular corotational twin screw extruder with a temperature profile between of 240–260 °C, a screw rate of 250 rpm, and a feed rate of 2.5 kg/h. The screw profile was configured with distributive and dispersive mixing elements, as illustrated in Figure 1. The final content of ZnO in the compound was 1% wt. Neat PET was submitted to similar compounding parameters for comparison purposes. Specimens were coded as PET and PET/ZnO 1% for neat PET and composites with 1% wt of ZnO, respectively.

### 2.3. Injection Molding

Tensile test bars were injection molded in an Arburg Injector, Model Allrounder 207C Golden Edition (ARBURG Technology Center, Kaiserstr, Radevormwald, Germany), according to ASTM D638 [16]. The injection molding parameters are presented in Table 1.

The injected specimens presented some whitening regions due to crystallization that developed during cooling. Aiming to avoid testing specimens with uncontrolled microstructures which could lead to unreliable results, all specimens were heated at 120 °C for 4 h to promote cold crystallization. 

### 2.4. Characterization

X-ray diffraction (XRD) analysis was conducted with injection molded samples in a SHIMADZU XRD-7000 (Shimadzu Corporation, Tokyo, Japan) using copper K_α_ radiation, 40 KV voltage, 30 mA current, and a scanning rate of 2/min with 2θ ranging from 5 to 80°. 

Differential Scanning Calorimetry (DSC) analyses were performed in a DSC-Q20 from TA Instruments (New Castle, DE, USA) using approximately 5 mg in sealed aluminum pans. Specimens were heated/cooled/reheated from 30 to 300 °C with a heating rate of 10°/min in nitrogen atmosphere with a gas flow rate of 50 mL/min.

The effect of NaOH and ZnO on the fractional crystallization (or relative crystallinity) and fractional fusion (or relative fusion) of PET was computed as a function of time by integration:

(1) $$x(t)=\frac{1}{E_{0}}\int_{t_{1}}^{t}\left|J(t^{\prime })-J_{0}(t^{\prime })\right|dt^{\prime }$$

where *J*_0_ is the virtual base line during the event (straight line in the present case) and *E*_0_ is the total latent heat of the phase change:

(2) $$E_{0}=\int_{t_{1}}^{t_{2}}\left|J(t)-J_{0}(t)\right|dt$$

where *t*_1_ and *t*_2_ are the initial and final times. The rate of the phase change (crystallization or fusion) *c* is:

(3) $$c(t)=\frac{dx}{dt}=\frac{\left|J(t)-J_{0}(t)\right|}{E_{0}}$$

from which the peak (maximum) and average crystallization (or fusion) rates were calculated. The fractional crystallization may be expressed as a function of temperature (*T*), knowing the linear relationship between time and temperature during the event: *T* = *T*_1_ + *ϕ* (*t* − *t*_1_), where *T*_1_ is the sample temperature at the starting point *t*_1_, *τ* = *t* − *t*_1_ is the time measured from the onset of the event, and *ϕ* = *dT*/*dt* is the (constant) rate of heating or cooling during the event. 

Thermogravimetry (TG) was performed using a Q20 calorimeter from TA Instruments. Specimens were heated from 30 to 600 °C with a heating rate of 10 °C/min in nitrogen atmosphere with a gas flow rate of 50 mL/min.

Tensile testing was performed with injected specimens, according to ASTM D638, using a universal test machine EMIC DL 2000 (EMIC, São Paulo, Brazil) with a crosshead speed of 50 mm/min, at room temperature (~23 °C). An aggressive fluid NaOH solution with a concentration of 3M was applied to the molded surfaces of the specimens during the test using a cotton pad. Reported data are averages of six specimens.

Scanning electron microscopy (SEM) images were captured from the specimen fractured surfaces. A Shimadzu SSX-550 Superscan (Tokyo, Japan) scanning electron microscope was used with a voltage of 30 kV under high vacuum. Prior to SEM analysis, the fracture surfaces were gold coated to avoid charge accumulation.

## 3. Results and Discussions

**Figure 2:**
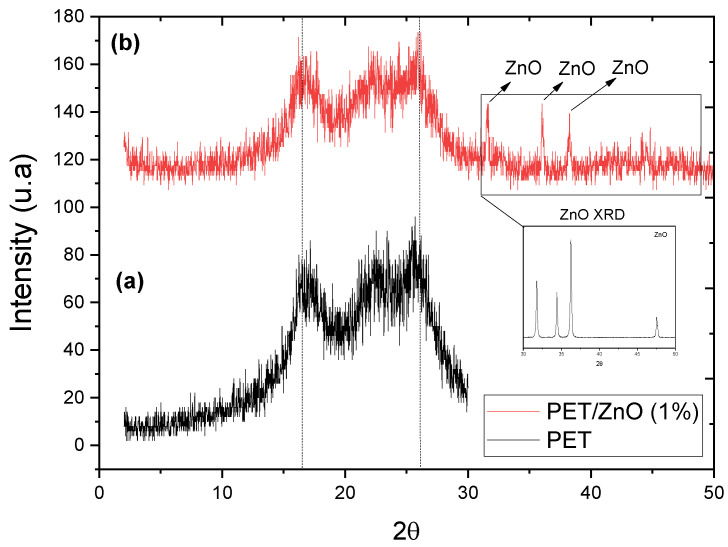
X-ray diffractograms of PET (**a**) and PET/ZnO 1% (**b**).

The X-ray diffractograms of the as-molded PET and PET/ZnO 1% samples given in Figure 2 indicate that a crystalline phase was present, together with amorphous regions. Neat PET presented two well-defined peaks at 2θ = 16.9° and 2θ = 26.2° due to the diffraction of the planes (010) and (100), as already reported [17,18]. These peaks are characteristic of the PET crystalline phase α, which has triclinic structure with parameters a = 4.56 Å, b = 5.94 Å and c = 10.75 Å, as well as angles between the axes 98.5°, 118°, and 112° [18,19]. The addition of 1% ZnO to PET was successful, since the XRD presented ZnO crystalline peaks. The verified PET/ZnO 1% displayed the same crystalline pattern as neat PET, that is, the addition ZnO did not impose structural modification on the PET matrix. 

Even though the injection molding conditions were chosen to produce only amorphous material (like the injection mold temperature set at 10 °C), some ordering did occur, possibly due to a nucleating action of the ZnO particles [19,20]. This caused some whitening of the test bars, which is also an indication of crystallization. Aiming to provide specimen homogeneity, both PET and PET/ZnO 1% specimens were submitted to a thermal treatment procedure at 120 °C for 4 h, allowing cold crystallization to take place [19] prior to further procedures. The influence of ZnO on PET crystallization will be discussed later, during the discussion of the DSC analyses. The crystallinity of PET might have an influence on the ESC behavior, as reported before for PET [12] and PHB [20].

**Figure 3:**
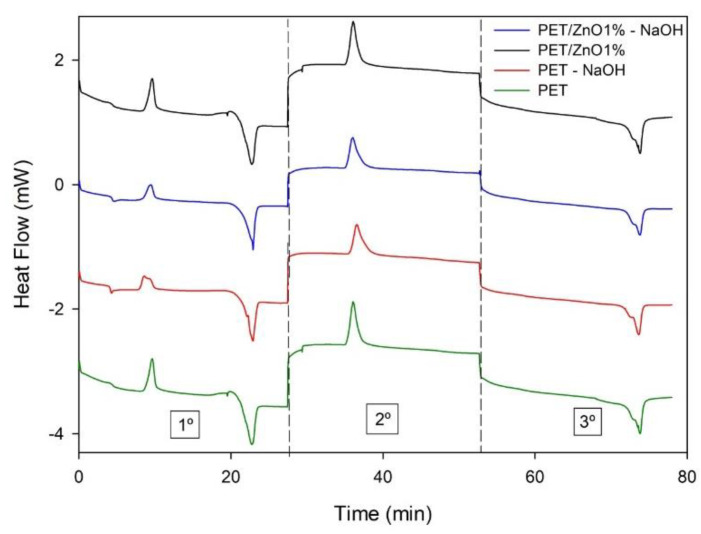
DSC scans of the whole thermal cycle—first heating (1°), cooling (2°), and second heating (3°). Compounds indicated. Heating/cooling/reheating rates of 10 °C/min.

Figure 3 displays DSC scans recorded during the experimental thermal cycle applied in this work, in which an exothermic and an endothermic peak were observed during the first heating (1°) due to the cold crystallization and melting of PET, respectively. It is worth noting that even after being subjected to thermal aging, some macromolecular chains that were still in the amorphous state were ordered during heating in the DSC. However, the cold crystallization peak of PET/ZnO was less intense than that for neat PET, indicating that most of the crystallization occurred between the injection cooling and thermal aging cycles [21]. Figure 3 also shows that the cold crystallization peak of PET/ZnO under NaOH effect is less intense and more complex, suggesting different crystallization mechanisms. 

**Scheme 1:**
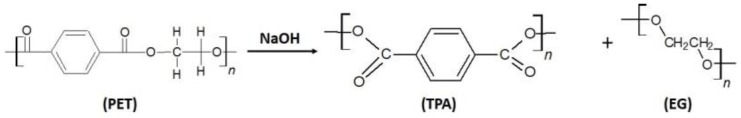
Suggested PET breakdown molecules upon contact with NaOH.

The presence of NaOH may cause a chemical reaction in the polymer chains of PET, as shown in Scheme 1, leading to a reduction in molecular sizes, as observed before [11]. Additionally, under NaOH effect, hydrolysis may take place in PET, also yielding lower macromolecular chains as well as monomers and oligomers. Therefore, these chains of different sizes tend to widen the crystallite sizes and perfections, promoting the formation of complex peaks. Complex fusion peaks were also seen during the second heating, mostly due to distinct morphologies and/or perfection.

**Figure 4:**
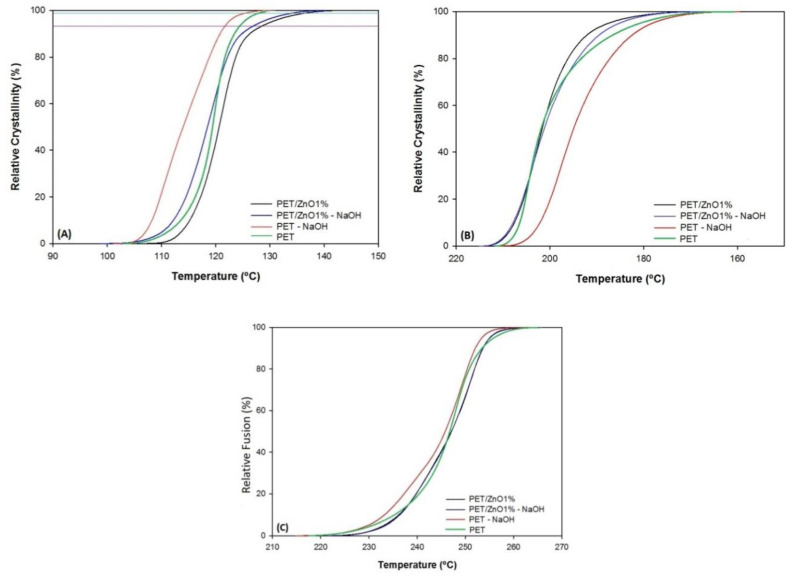
“S” plots. (**A**) Relative Cold Crystallinity. (**B**) Relative Melt Crystallinity. (**C**) Relative Fusion. Compounds indicated. Heating/Cooling/Reheating rate of 10°/min.

From the DSC scans, the relative crystallinities were calculated; the results are given in Figure 4 for both cold crystallization and crystallization from the melt. Figure 4 also shows the relative fusion obtained from the DSC peaks using Equations (1) and (2). Under NaOH effect, the cold crystallization of PET was shifted to lower temperatures, possibly due to a lower molar mass. Related to the addition of ZnO to PET, the cold crystallization of PET/ZnO 1% took place at lower temperatures. It is therefore suggested that ZnO acts as heterogeneous centers, decreasing surface energy for crystallite nucleation and growth, and hence improving the crystallization mechanisms of PET. Additionally, the presence of ZnO seems to reduce the effect of NaOH, indicating that its presence may stabilize the molecular structure, as the plot of PET/ZnO 1%-NaOH is displayed at a higher temperature than that of PET-NaOH. Similar behavior was observed during melt crystallization. Regarding the melting behavior, PET-NaOH melted at a lower temperature, suggesting the existence of smaller and/or less perfect crystals. A sigmoidal shape was also observed, which is indicative of a continuous phase transition.

**Figure 5:**
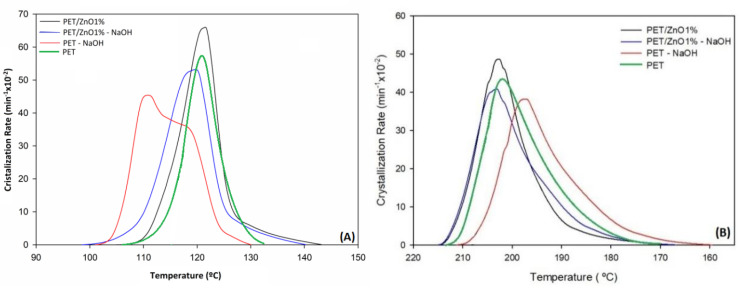

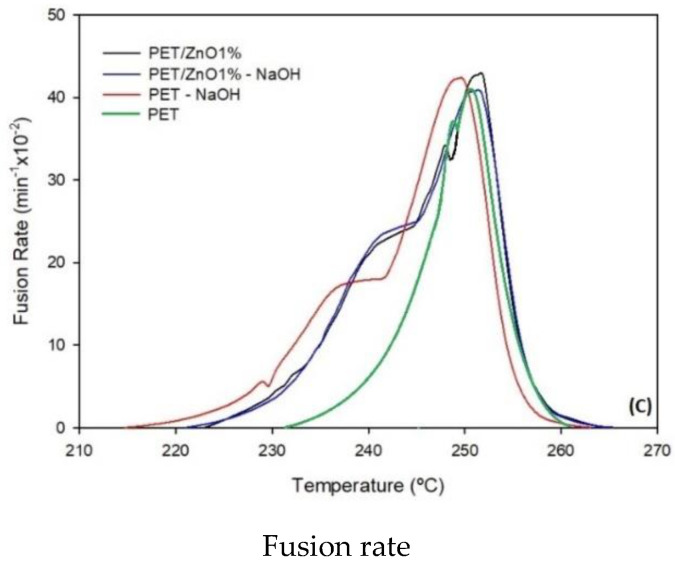
Crystallization and fusion rates according to temperature for various compounds. (**A**) Cold crystallization. (**B**) Melt crystallization. (**C**) Fusion.

Plots of crystallization and fusion rates, calculated using Equation (3), are graphically presented in Figure 5. PET and PET/ZnO presented bell shaped plots whereas, upon exposure to NaOH, more complex curves were obtained, mostly due to the macromolecular weight dispersity leading to distinct rates for crystallizing. This might be true for both heating and cooling, which may be related to the complexity of the crystallite morphologies. The thermodynamic and kinetic parameters obtained from DSC scans are presented in Table 2.

**Figure 6:**
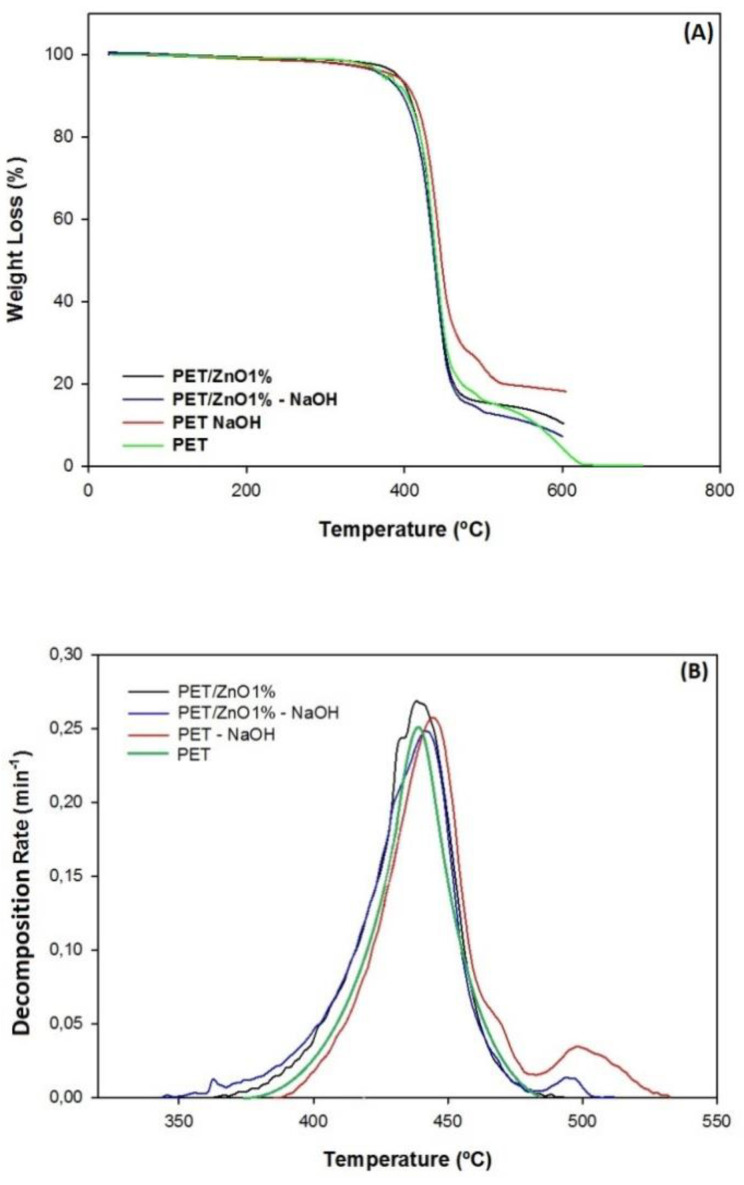
TGA plots of the compounds under study. (**A**) Weight loss. (**B**) Decomposition rate.

Figure 6 shows TGA plots of the investigated compounds, and Table 3 presents the kinetic parameters calculated from them. All compounds presented a single step of weight loss, with little influence of ZnO and NaOH. Related to the degradation rate of PET, according plots on Figure 6B, all investigated compounds presented a similar trend, with *T*_max_ ~435 °C [22].

## 4. Mechanical Properties

**Figure 7:**
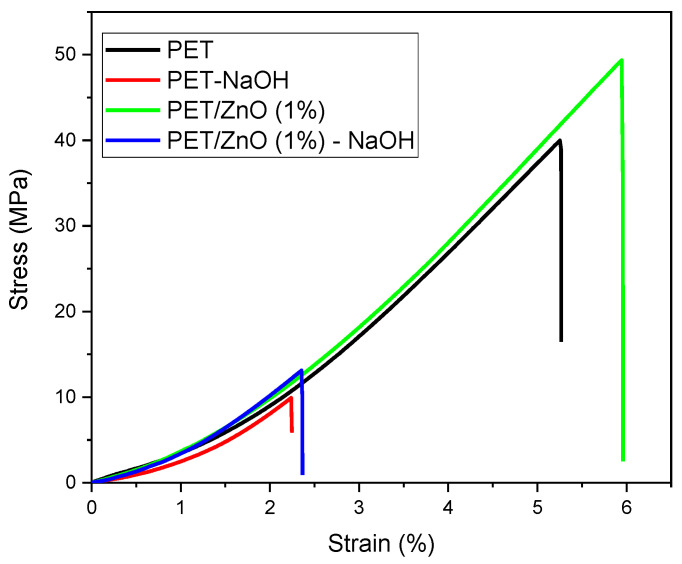
Stress versus strain plot of tested compounds.

NaOH in aqueous solution is a stress cracking agent of PET [7,10] that causes surface cracking and a reduction in mechanical properties. This is evidenced in Figure 7 and Table 4. 

Figure 7 presents stress versus strain plots of the tested compounds, about which two observations can be made:

- Related to the action of NaOH, it is clearly seen that PET lost its ability to withstand high stress, fracturing at lower strain; see the PET and PET-NaOH plots.
- Related to the action of ZnO, PET presented not only higher stress but also higher strain ability; see the PET and PET/ZnO plots. Upon exposure to NaOH, ZnO provided the PET with the ability to withstand higher stress and strain, as presented in the PET–NaOH and PET/ZnO (1%)–NaOH plots.

According to Table 4, NaOH reduced both the force and maximum elongation of PET, whereas ZnO provided a protective action against this harmful agent. The cracks formed at the sample surface act as stress raisers that propagate into the underlying material, causing failure due to brittleness [19]. If the molar mass was reduced, the effect was even more pronounced than when just ESC took place. The presence of ZnO caused some increase in tensile properties, due to a higher degree of crystallinity, but did not prevent the stress cracking deteriorating effects.

**Figure 8:**
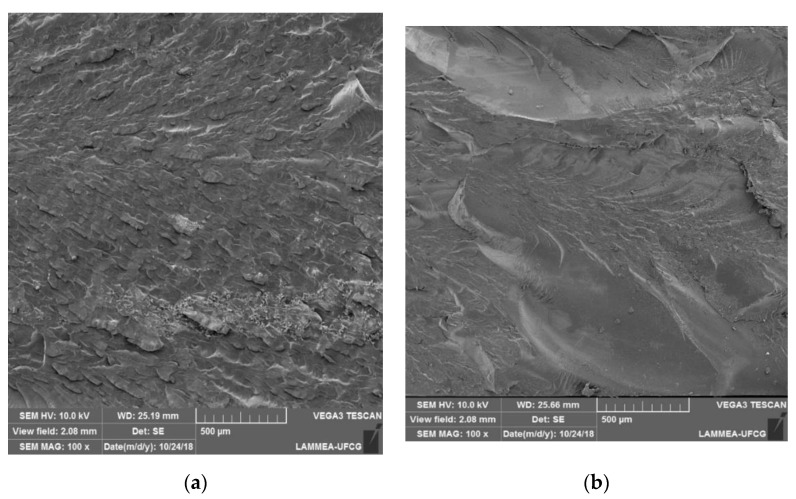
SEM images of PET (**a**) and PET–NaOH (**b**).

Figure 8 shows SEM images captured from the fracture surface of PET (a) and PET NaOH (b). In Figure 8a, a rough surface is clearly observed, suggesting a ductile fracture with high energy consumption during the failure process. This differs from the fracture in the presence of NaOH (Figure 8b), where a much smoother surface was observed, indicating a brittle fracture with lower energy consumption. These observations are consistent with the results shown in Table 4.

**Figure 9:**
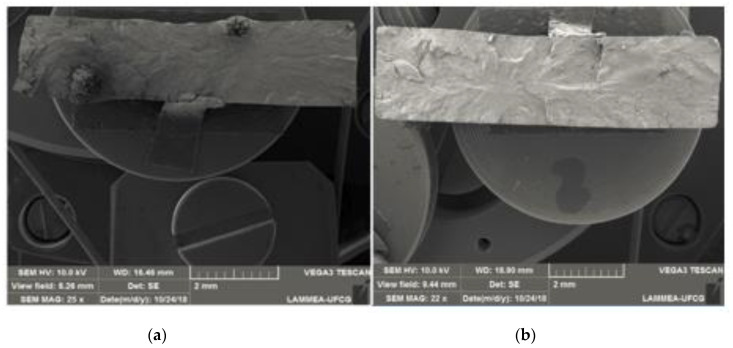
SEM images of PET/ZnO 1% (**a**) and PET/ZnO 1%-NaOH (**b**).

Figure 9 shows the fracture surfaces of PET with ZnO and the effect of NaOH. The precise difference between these two conditions on fracture behavior is unclear; nevertheless, it is reasonable to suggest that Figure 9b shows larger smooth regions due to the higher fragility, which is consistent with the tensile data presented in Table 4.

## 5. Conclusions

The present study aimed to evaluate the stress cracking effects caused by NaOH solutions on the thermal and tensile properties of PET and PET/ZnO composites. The results showed that NaOH did not significantly change the thermal stability PET, but that the cold and melt crystallization, as well as the melting behavior, changed by its influence. Cold crystallization was observed at lower temperatures, whereas more complex peaks were obtained during melting, suggesting the presence of smaller and less perfect crystals. The mechanical properties of PET were reduced when NaOH was applied during tensile testing, indicating failure by stress cracking. On the other hand, ZnO not only improved PET crystallization, mostly due to heterogeneous centers improving crystallinity, but also offered some protection against the harmful effects of NaOH.

## Figures and Tables

**Table 1:** Injection parameters.

Parameters	
Injection pressure (bar)	1150
Temperature (°C)	280 in all zones
Injection Flow (cm^3^/s)	6
Mold temperature (°C)	10
Cooling time inside the mold (s)	25
Holding pressure (bar)	500

**Table 2:** Thermodynamic and kinetic parameters of crystallization and fusion obtained from DSC scans.

Compound		*T*_0.1%_ (°C)	*T*_50%_ (°C)	T_99%_ (°C)	*c_max_* (min^−1^ × 10^−2^)	*c*_0.1–99.9_ (min^−1^)	*T_max_* (°C)	τ_½_ (min)
Cold Crystallization	PET/ZnO1%	108.36	120.61	141.27	0.01517	0.0548	32.90	0.02
	PET/ZnO1%–NaOH	101.64	118.47	138.44	0.01878	0.0613	36.80	0.03
	PET–NaOH	103.06	113.97	128.56	0.02202	0.0425	25.51	0.02
Melt Crystallization	PET/ZnO1%	213.32	201.68	171.10	0.02052	0.0704	42.22	0.02
	PET/ZnO1%–NaOH	213.56	201.25	170.46	0.02453	0.0718	43.10	0.02
	PET–NaOH	209.02	194.98	163.31	0.02616	0.0762	45.71	0.02
Fusion	PET/ZnO1%	224.63	247.11	262.53	0.02361	0.0632	37.90	0.04
	PET/ZnO1%–NaOH	223.46	247.09	262.33	0.02446	0.0648	38.88	0.04
	PET–NaOH	217.85	245.81	260.17	0.02360	0.0705	42.32	0.05

*T*_0.1%_ (°C) is the temperature for 0.1% of crystallized (or molten) material (a good estimation of the event initial point). *T*_50%_ (°C) is the temperature for 50% of crystallized (or molten) material. *T*_99.9_ (°C) is the temperature for 99.9% of crystallized (or molten) material (a good estimation of the event final point). *c_max_* (min^−1^) is the maximum crystallization (or fusion) rate, i.e., at the peak. *c*_0.1–99.9_ is the crystallization (or fusion) rate between 0.01 and 99.9 phase transformation. *T*_max_ (°C) is the temperature at the crystallization (fusion) peak. τ_½_ (min) half crystallization (or fusion) time (time to reach 50% crystallized or molten material).

**Table 3:** Thermodynamic and kinetic parameters of decomposition computed from TG scans.

Compound	*T*_0.1%_ (°C)	*T*_50%_ (°C)	*T*_99%_ (°C)	*d_max_* (min^−1^)	*d*_0.1–99.9_ (min^−1^)	*T_max_* (°C)	τ_½_ (min)
PET/ZnO1%	370.38	435.71	482.13	3.72439	11.105	111.76	6.5
PET/ZnO1%–NaOH	353.61	435.01	500.39	4.01606	14.554	146.78	8.09
PET–NaOH	392.45	442.72	527.61	3.87747	13.427	135.16	5.02

*d_max_* (min^−1^) is the maximum decomposition rate, i.e., at the peak.

**Table 4:** Tensile properties of PET and PET/ZnO under effect of NaOH.

Compounds	Force (N)	Elongation at Break (%)
PET	1649.8 ± 171.4	4.8 ± 0.8
PET–NaOH	471.8 ± 63.8	2.1 ± 0.4
PET/ZnO1%	2011.1 ± 209.6	5.5 ± 0.3
PET/ZnO1%–NaOH	675.2 ± 73.8	2.5 ± 0.4
